# Enhanced Long-Term Reliability of Seal DeltaSpot Welded Dissimilar Joint between 6061 Aluminum Alloy and Galvannealed Steel via Excimer Laser Irradiation

**DOI:** 10.3390/ma14226756

**Published:** 2021-11-09

**Authors:** Sung-Min Joo, Young-Gon Kim, Young-Jin Kwak, Dong Jin Yoo, Chang-U Jeong, Jeshin Park, Min-Suk Oh

**Affiliations:** 1 Department of Naval Architecture and Ocean Engineering, Chosun University, Gwangju 61452, Korea; joo@chosun.ac.kr; 2 EV Component & Materials R&D Group, Korea Institute of Industrial Technology (KITECH), Gwangju 61012, Korea; ygkim1@kitech.re.kr; 3 POSCO Technical Research Laboratories, Gwangyang 545875, Korea; sauri777@naver.com; 4 Department of Energy Storage/Conversion Engineering of Graduate School, Jeonbuk National University, Jeonju 54896, Korea; djyoo@jbnu.ac.kr; 5 Department of Life Science, Hydrogen and Fuel Cell Research Center, Jeonbuk National University, Jeonju 54896, Korea; 6 Division of Advanced Materials Engineering and Research Center for Advanced Materials Development, Jeonbuk National University, Jeonju 54896, Korea; joseph94@jbnu.ac.kr (C.-U.J.); parkjs@jbnu.ac.kr (J.P.)

**Keywords:** dissimilar joint, galvanic corrosion, adhesive bonding, excimer laser irradiation

## Abstract

Structural-adhesive-assisted DeltaSpot welding was used to improve the weldability and mechanical properties of dissimilar joints between 6061 aluminum alloy and galvannealed HSLA steel. Evaluation of the spot-weld-bonded surfaces from lap shear tests after long-term exposure to chloride and a humid atmosphere (5% NaCl, 35 °C) indicated that the long-term mechanical reliability of the dissimilar weld in a corrosive environment depends strongly on the adhesive–Al6061 alloy bond strength. Corrosive electrolyte infiltrated the epoxy-based adhesive/Al alloy interface, disrupting the chemical interactions and decreasing the adhesion via anodic undercutting of the Al alloy. Due to localized electrochemical galvanic reactions, the surrounding nugget matrix suffered accelerated anodic dissolution, resulting in an Al6061-T6 alloy plate with degraded adhesive strength and mechanical properties. KrF excimer laser irradiation of the Al alloy before adhesive bonding removed the weakly bonded native oxidic overlayers and altered the substrate topography. This afforded a low electrolyte permeability and prevented adhesive delamination, thereby enhancing the long-term stability of the chemical interactions between the adhesive and Al alloy substrate. The results demonstrate the application of excimer laser irradiation as a simple and environmentally friendly processing technology for robust adhesion and reliable bonding between 6061 aluminum alloy and galvannealed steel.

## 1. Introduction

Energy conservation and climate change have greatly influenced the development of advanced materials and processing technologies for a myriad of industrial applications [1,2,3]. The adoption of lightweight hybrid structures in automotive, aircraft, and marine industrial components can reduce body weight and consequently the greenhouse gas emissions and fuel consumption of machinery and vehicles [4,5]. Since joining is one of the most important manufacturing technologies, achieving reliable welding between dissimilar high-tensile strength steel and lightweight materials, such as aluminum, magnesium, and fiber-reinforced composites, has become significantly important [6,7,8]. In the automotive industry, resistance spot welding has been most employed in metal sheet assembly due to its automation adaptability for high-volume production, superior joint structural performance, and easy control [9,10]. However, the practical use of fusion-based resistance spot welding between Al alloys and steel has been hampered by difficulties in welding control arising from the large differences in the physical properties of each material, such as low melting temperature and electrical conductivity [11,12,13]. Furthermore, the extremely low solubility of Fe in Al is known to cause brittle intermetallic phases such as Fe*_x_*Al_100−*x*_ at the welding interface, which worsen the mechanical properties of the joints [14,15]. Moreover, when two dissimilar metals in an overlap configuration come into electrical contact in the presence of a corrosive electrolyte, galvanic and perforation corrosion can become additional major problems that accelerate corrosion of the welded joint [16,17].

To prevent degradation, sealing with the supplementary application of insulating structural adhesives, combined with spot welding, has been proposed to not only prevent galvanic corrosion by sealing the weld joint but also to offer benefits such as improved fatigue resistance and vibrational damping for structural frames and components [18,19]. However, the mechanical characteristics of dissimilar joints with adhesives can deteriorate under prolonged exposure to a humid and corrosive environment due to the penetration of corrosive electrolytes such as water molecules and chloride ions into the cut-edge surface, resulting in anodic undercutting and the loss of adhesive strength [20,21]. In the aerospace industry, Al alloys are subjected to anodization surface pretreatment using phosphoric or chromic acids to prevent adhesion degradation [22,23]. A toughened adhesive/substrate interface has also been reported to induce a low oxygen permeability and thus reduce cathodic delamination [24]. In addition, studies on Al surface preparation prior to adhesive bonding using laser irradiation [25], atmospheric plasma [26], immersion in sodium hydroxide [27], and electrospraying with hydrophobic silica fumes [28] to prevent degradation of the adhesive bond strength have been conducted. However, the relationship between corrosion and the degradation of the mechanical properties of adhesive-assisted spot-welded dissimilar joint structures has not yet been fully clarified. Moreover, for automotive applications with high production volumes, there is a great need for simpler, more economical, and environmentally friendly processing technologies to improve the bond strength of welded joints.

In the present study, the DeltaSpot welding (DSW) method, a versatile spatter-free welding technology that protects the contact surface of an electrode from Al residue contamination using process tape, was employed to achieve dissimilar welding between Al6061 alloy and galvannealed (GA) steel plate with favorable weldability and mechanical properties. To improve the long-term reliability, the joints were sealed by supplementary insulating structural adhesives. In addition, to promote strong interfacial interactions between the adhesive polymer and substrate, a highly efficient dry surface treatment (KrF pulsed excimer laser treatment (PLAT)) was performed to modify the surface chemistry and morphology of the Al6061-T6 alloy. The effect of PLAT on the bond strength of the joints after accelerated corrosion tests was studied to understand the relationship between corrosion and the bond strength degradation of adhesive-assisted DSW dissimilar metal weldments.

## 2. Materials and Methods

### 2.1. Materials

The substrates used in this work were 1-mm-thick Al6061-T6 alloy plates containing Mg–Si–Cu alloying elements and sheets of GA high-strength low-alloy steel (GA HSLA 340YC, POSCO Co., Ltd., Pohang, Korea), a precipitation-hardened steel with a high yield strength and impact resistance. The nominal chemical compositions and mechanical properties of the base materials were measured by using an X-ray fluorescence analysis and an Instron-4208 universal testing machine, and the results are presented in Table 1 and Table 2, respectively. The high-strength 340YC steel was immersed in a molten Zn bath with an Al content of 0.12 wt% and then subjected to an annealing cycle at 500 °C for 3 s to enable the reaction between Zn and Fe. Inductively coupled plasma analysis indicated that the GA had a coating weight of 55 g/m^2^ on one side and contained approximately 12.9 wt% Fe, as shown in Table 1.

### 2.2. Excimer Laser Irradiation and Adhesive Application

**Figure 1:**
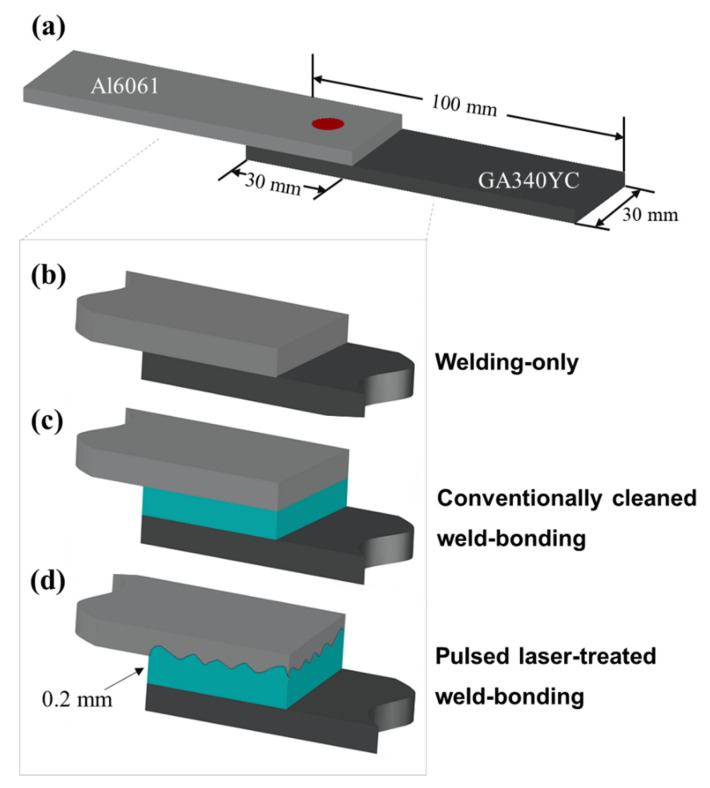
Schematics of (**a**) the assembled single lap shear specimens and the specimen arrangements of joints: (**b**) WO, (**c**) CWB, and (**d**) PWB.

To prevent the penetration of corrosive electrolytes into the overlapped sheets, a commercial thermosetting epoxy resin-based structural adhesive (SA-1402D, Pusan, Bokwang Corp.) was applied as a sealant to the Al alloy substrate. Prior to adhesive application, the surfaces of the Al alloy were ultrasonically degreased by immersion in acetone, methanol, and deionized water for 5 min each, followed by N_2_ gas blowing; this procedure hereafter is referred to as conventional cleaning, and the samples are referred to as conventionally cleaned. For the laser-treated samples, the Al alloy surface was first conventionally cleaned and then treated with a pulsed excimer laser using a homogenized KrF excimer laser (Lambda Physik LPX205, Göttingen, Germany); these samples are referred to as PLATed. The use of a beam homogenizer yielded a uniform irradiation profile of 5 × 5 mm^2^ on the substrate. The excimer laser has a wavelength of 248 nm and a pulse duration of 25 ns with an energy density of 300 mJ/cm^2^ as measured by an energy power meter. The Al alloys were exposed to the laser for 5 min at a repetition rate of 5 Hz in an N_2_ atmosphere. The adhesive-applied Al alloys were then covered by GA steel sheets and mechanically pressed at a pressure of approximately 3–5 bar. During the adhesive application and pressing procedure, the 0.2 mm thick adhesive was uniformly adjusted by steel spacers. The assembled single lap shear specimens, as shown in the schematics in Figure 1, consisted of two single panels (80 mm × 10 mm) joined with a 25 mm overlap in accordance with the ISO 14273:2016 standard. Figure 1b–d schematically show spot welding of the sample without the adhesive, with the adhesive, and with the adhesive applied after excimer laser treatment, respectively.

### 2.3. DSW and Accelerated Corrosion Test

**Figure 2:**
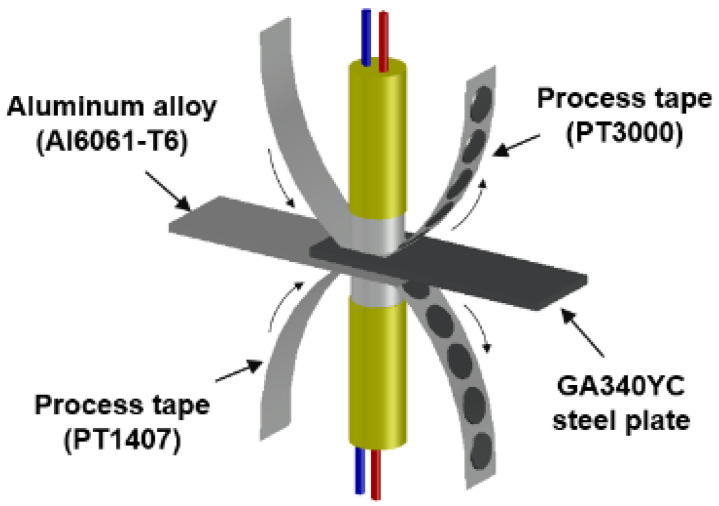
Schematic of the DeltaSpot welding simulator.

Thermo-compensated DSW was employed to prevent surface oxides from adhering to the welding electrodes by balancing the thermal flow between dissimilar target materials [29,30]. A Cr–Ni-based process tape (PT3000) and a steel-based process tape (PT1407) were chosen as contact electrodes for the Al6061-T6 and GA340YC substrates, respectively, as shown in the schematic of the DSW simulator in Figure 2. The DSW samples treated with applied adhesive after conventional cleaning and after PLAT are hereafter referred to as conventionally cleaned weld-bonding (CWB) and PLATed weld-bonding (PWB), respectively. The spot-welded sample without adhesive is referred to as welding only (WO). DSW conditions such as the input current, welding pressure, and current injection time were adjusted to control the heat injection and thus ensure that all weldments had similar nugget diameters as measured by peeling tests of the welded coupons. The welding conditions used in this study are listed in Table 3, which shows that a higher welding pressure and injection current were required under same duration for the samples with the insulating adhesive. After DSW, the adhesives in the weld-bonded samples were hardened at 180 °C for 30 min in an air circulating furnace. To assess the inhibitory effect of sealed DSW on the corrosion resistance, all the samples were subjected to a salt spray test (SST, Erichsen 606/2000) in accordance with the ASTM B-117 standard using an NaCl concentration of 5 wt%, temperature of 35 °C, relative humidity of >98%, and a flow rate of 1.8 mL/h. Tensile tests on the samples, which were maintained in the SST chamber for 1500 h, were then performed.

### 2.4. Characterization

The microstructures of the samples were observed using an optical microscope (OM) (DM2500, Leica, Wetzlar, Germany), field-emission scanning electron microscopy (FE-SEM) (JSM-7100F, JEOL, Tokyo, Japan) and transmission electron microscopy (TEM) (JEM-2200FS, JEOL). The chemical composition of each microstructure was determined by energy-dispersive X-ray spectroscopy (EDS), and the microstructural phases were investigated using X-ray diffraction (XRD) (XRD-6100 with a Cu Kα X-ray source, Shimadzu, Kyoto, Japan). A Vicker’s microhardness tester (HM-112, Matsuzawa, Akita, Japan) was used to measure the hardness of the joints according to the E92-17 standard. The morphological changes and chemical bonding states of the conventionally cleaned and PLATed Al alloys were characterized using a high-resolution optical 3D surface profiler (NS-3500, Nanoscope, Daejeon, Korea) and X-ray photoelectron spectroscopy (XPS) (ESCALAB 250 XPS, Massachusetts, USA), respectively. The XPS measurements were carried out using a monochromatic Al Kα X-ray source (1486.6 eV) in an ultrahigh vacuum system with a base chamber pressure of 10^−9^ Torr. Before measurement, the conventionally cleaned Al alloys were stored for 3 d in a vacuum desiccator at a pressure of 10^−4^ mmTorr. Electrochemical measurements were carried out using a three-electrode setup in 3.5% NaCl solution to evaluate the corrosion resistance through pitting potential test. The exposed dimension of the working electrode was 10 × 10 mm^2^. A saturated calomel electrode was used as the reference electrode. The bond strengths of the samples before and after exposure to the accelerated corrosion test conditions were evaluated in accordance with KS B 0802:2003 (method of tensile test for metallic materials) via single lap shear tests using Shimadzu AG-X universal testing equipment operated at a cross-head velocity of 1 mm/min. Three samples were tested under each set of conditions for reproducibility.

## 3. Results and Discussion

**Figure 3:**
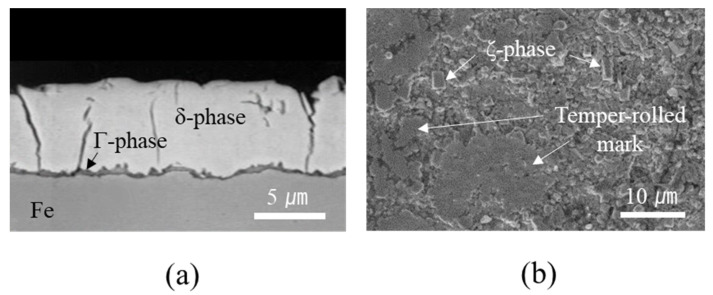
Microstructure of GA coating: (**a**) cross-section and (**b**) surface.

**Figure 4:**
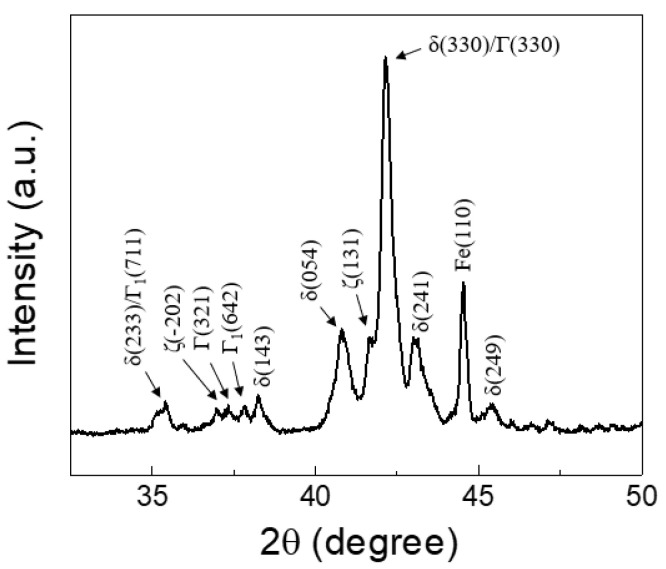
XRD spectrum of the GA coating.

Figure 3a shows the cross-sectional microstructure of the GA coating layer, illustrating an average coating thickness of 8.4 μm. Before Zn solidification in the GA process, Zn and Fe interdiffuse during thermal annealing and break down the Al–Zn–Fe inhibition layer, resulting in the formation of intermetallic compounds (IMCs) such as gamma (Γ_1_: Fe_5_Zn_21,_ Γ: Fe_3_Zn_10_), delta (δ: FeZn_7_), and zeta (ζ: FeZn_13_) phases [31,32]. As shown in Figure 3a, the GA coating layer in this study consisted of two distinct phases: a thin interfacial layer and a thick overlayer. Based on quantitative EDX measurements, Fe compositions of the interfacial and overlayers corresponding to the Γ (Γ_1_ and Γ) and δ phases were evaluated to be 15.9–18.4 wt% and 8.1–10.3 wt%, respectively. Although, as shown in Figure 3b, a small amount of rod-like monoclinic ζ phase was observed on the surface, the XRD spectra (Figure 4) also revealed that the GA coating consisted predominantly of the δ and Γ phases, implying that the coating layer was over-alloyed. As shown in Figure 3a, cracks were also observed in the coating layer due to high brittleness of the Fe–Zn IMCs. The formation of high-Fe-content intermetallic alloy structures increased the surface roughness (*R*_rms_: ~2.8 μm) compared with that of the non-alloyed conventional Zn coating layer (*R*_rms_: ~1.2 μm), although temper-rolled marks on the GA surface (Figure 3b) indicated that the surface roughness of the steel was regulated by temper rolling.

**Figure 5:**
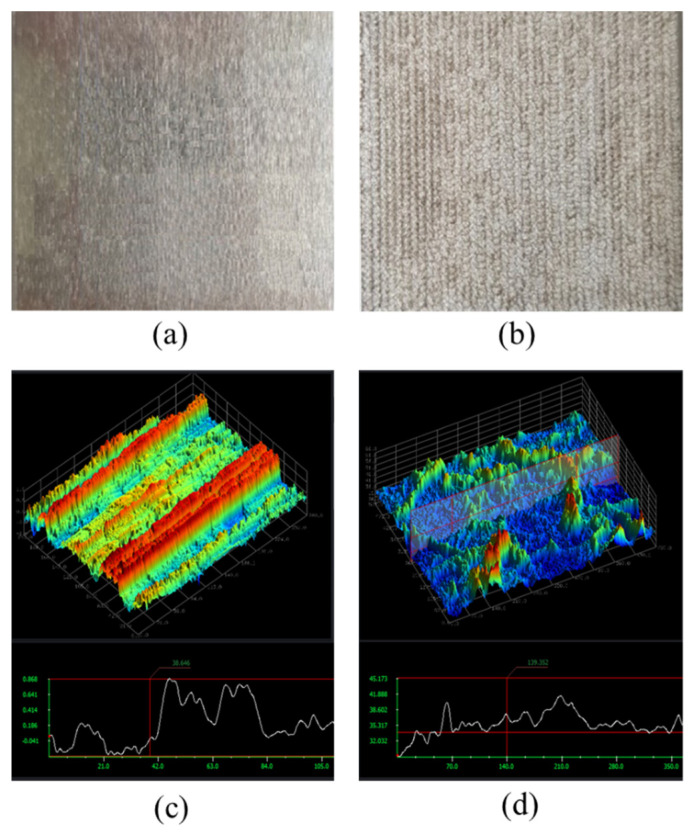
Three-dimensional surface profile images of Al6061 Al alloys (**a**,**c**) before and (**b**,**d**) after laser irradiation.

To improve the interfacial interaction between the adhesive and Al alloy, PLAT was performed on the surface of Al6061-T6. To observe the changes in the surface morphology of the Al alloy induced by laser irradiation, 3D surface profile images of Al6061-T6 before and after laser irradiation were obtained and are shown in Figure 5. The root-mean-square roughness (*R*_rms_) of the Al alloy was 0.3 and 3.25 μm before and after laser irradiation, respectively, indicating that the treatment ablated the surface elements and created a micro-rough surface topography.

**Figure 6:**
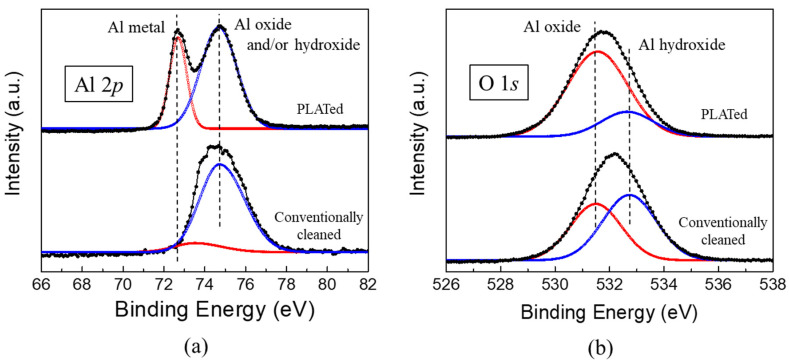
(**a**) Al 2p and (**b**) O1s spectra of Al6061 Al alloys before and after laser irradiation.

The changes in the surface chemistry of the Al6061-T6 substrate after PLAT were observed by XPS measurements. Figure 6a shows the Al 2p XPS spectra obtained from the surface of the Al alloy before and after laser irradiation. For the conventionally cleaned Al alloy, a single core-level peak was observed at 74.6 eV. However, after laser irradiation, another peak at a lower binding energy of 72.6 eV appeared. The relatively thin oxide layer (<80 Å) on Al alloys allows for the simultaneous observation of peaks corresponding to both Al oxides (and/or Al hydroxides) and the Al substrate in the Al 2p XPS spectra [33,34]. Therefore, the peak at 72.6 eV in the Al 2P spectrum is characteristic of the Al substrate, whereas that at 74.6 eV corresponds to an Al oxide and/or hydroxide layer. The thickness of the oxidic layer can be estimated from the relative peak intensity ratio between the oxide overlayer and Al substrate [35] by the following relationship:

$$d\left(Å\right)=\lambda_{0}\sin\theta \ln\left[\frac{N_{m}}{N_{0}}\frac{\lambda_{m}}{\lambda_{0}}+1\right],$$

$$d\left(Å\right)=24\ln\left(1.4\frac{I_{0}}{I_{m}}+1\right),$$

where *d* is the oxide thickness (Å); *N*_m_ and *N*_0_ are the volume densities of the substrate and oxide, respectively; λ_m_ and λ_0_ are the inelastic mean free paths of photoelectrons in the substrate and oxide (Å), respectively; *I*_m_ and *I*_0_ are the peak intensities of the substrate and oxide, respectively; θ is the electron take-off angle with respect to the sample surface. In this study, we used inelastic mean free path values of 24 and 22 Å for the Al oxide layer and Al substrate, respectively. The *N*_m_/*N*_0_ ratio was assumed to be 1.5 [36,37], and an electron take-off angle of 90° was used based on the sample/detector orientation in the XPS instrument. The thicknesses of the oxidic overlayers on the Al6061-T6 alloy before and after laser irradiation were calculated as approximately 73 and 22 Å, respectively, indicating that the treatment reduced the layer thickness.

The surfaces of Al alloys that have been exposed to the atmosphere are known to become hydrated to some extent due to the adsorption of H_2_O molecules. Therefore, it is believed that the oxidic component of the Al 2p spectrum shown in Figure 6a represents both Al oxide and hydroxide species [38,39]. The relative contents of Al oxide and hydroxide can be estimated according to the XPS O1s peak [33,40]. As shown in Figure 6b, deconvolution of the O1s spectrum indicated two core-level peaks corresponding to Al oxide (532.8 eV) and Al hydroxide (531.3 eV). Several monolayers of Al hydroxide or hydrated Al oxide, such as boehmite (AlO(OH)) and bayerite (Al(OH)_3_), form on top of the oxide layer from a corrosion reaction in a corrosive environment, which converts the dense native oxide layer to porous and hydrous species [41,42]. Figure 6b clearly illustrates that the intensity of the Al hydroxide peak markedly decreased after laser irradiation, implying that the corrosion-related Al(OH)*_x_* layer was not removed by the conventional cleaning process but was effectively removed by PLAT. Additionally, it should be noted that, upon exposure to ambient conditions, a thin Al oxide layer forms instantaneously even after PLAT.

**Figure 7:**
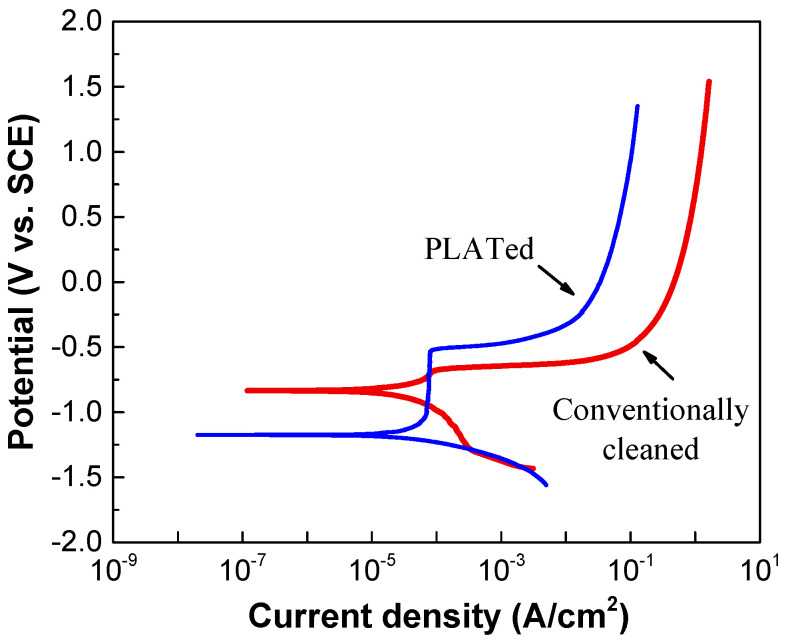
Potentiodynamic polarization curves of Al6061 Al alloys before and after laser irradiation.

Figure 7 shows the results of the electrochemical polarization measurements of the Al alloy before and after laser irradiation. As shown in Figure 7, the laser-irradiated sample showed lower corrosion current density. It is reported that this may be due to the presence of two dense oxide layers composed of nanocrystalline α-Al_2_O_3_ structures on top of the laser melted zone [43]. The pitting potential of the laser-irradiated sample was found to be slightly above the corrosion potential of the conventionally cleaned sample. Mechanisms of the behavior related to this are currently under investigation.

**Figure 8:**
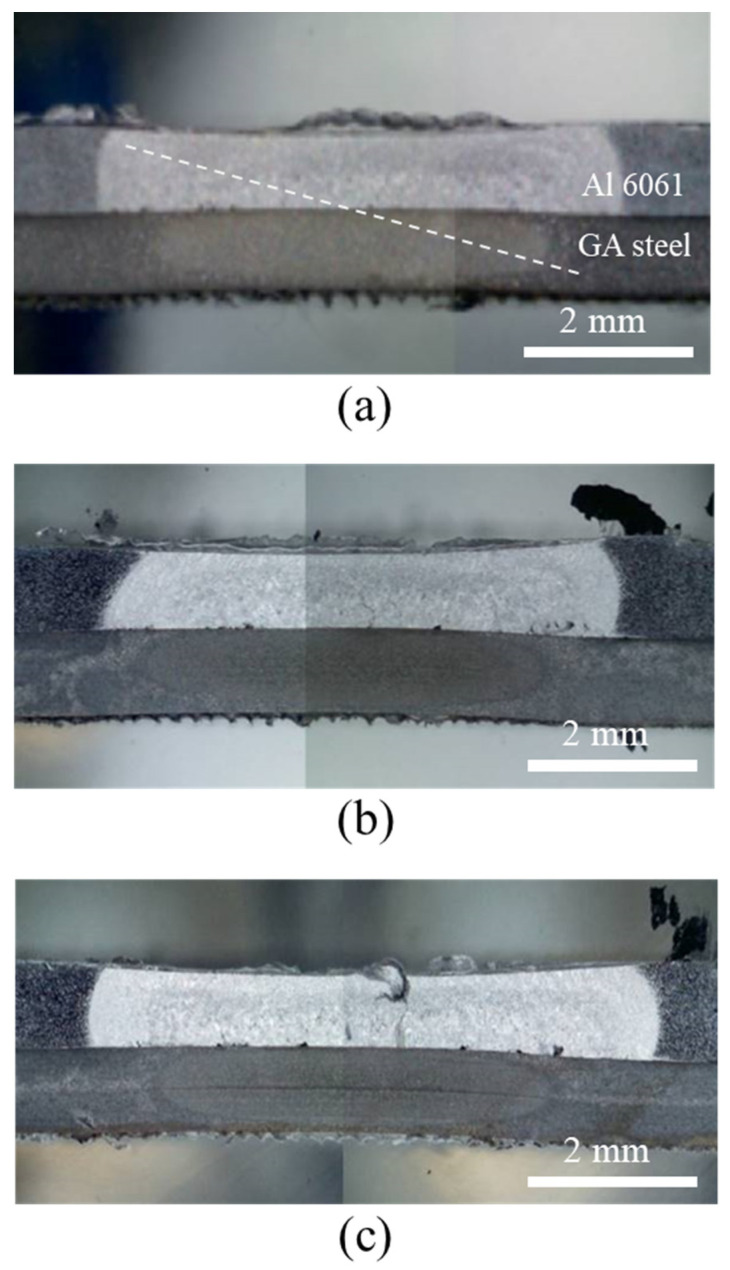
Cross-sectional OM images of joints after DSW: (**a**) WO, (**b**) CWB, and (**c**) PWB.

Figure 8 shows cross-sectional OM images of the joints after DSW. Despite balancing the thermal flow between the dissimilar target materials by inserting process tapes, the Al alloy and GA steel exhibited differently shaped nuggets and heat-affected zones (HAZs). This is likely due to the inherently different thermal conductivities of the two metals. The contact interface between two dissimilar metals hinders the heat flow such that heat is reflected and reemitted to the surroundings, and thus the Al alloy, with a higher thermal conductivity than Fe (Al 6061-T6: 167 W/(m∙K), Fe: 55–67 W/(m∙K)), has a larger HAZ. The Al6061 alloy was instantly melted by joule heating and deformed into a thinner shape by the electrode force. Some voids were observed in the Al alloy near the interface, which were particularly pronounced in the adhesive-applied specimens (Figure 8b,c), as discussed later.

**Figure 9:**
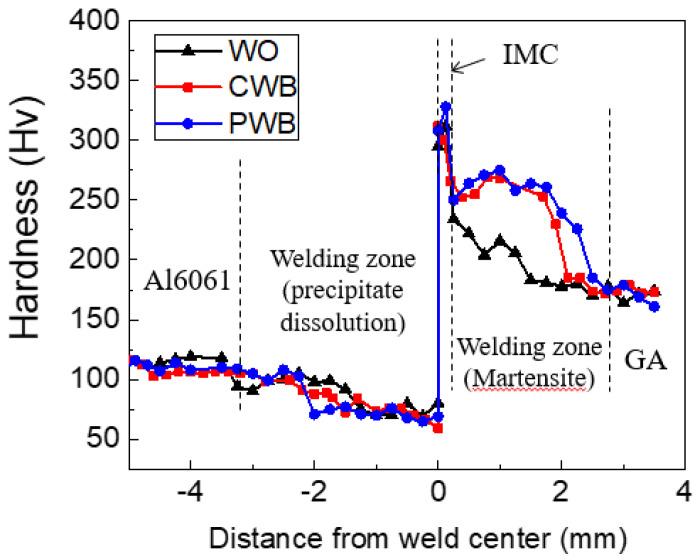
Hardness profiles along the transverse cross section marked by the dotted line in Figure 7a.

**Figure 10:**
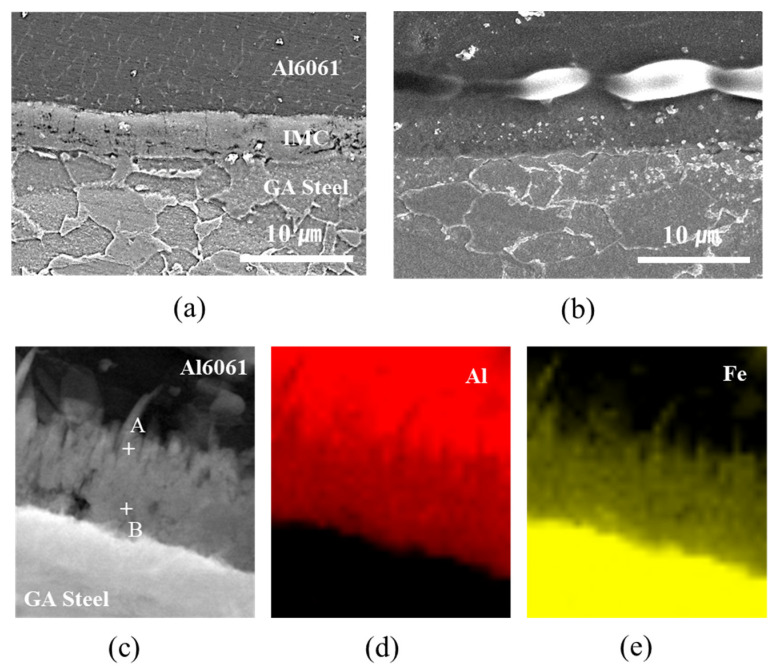
FE-SEM images of the microstructure of the joint interfaces of (**a**) WO and (**b**) CWB; (**c**–**e**) elemental mapping results of the interface measured by TEM joint.

The hardness profiles measured up to ±5 mm along the transverse cross-section marked by the dotted line in Figure 8a and are shown in Figure 9. The Fe–Al IMCs at the interfacial area exhibited the highest hardness. The microstructures of the joint interfaces observed by FE-SEM and TEM are shown in Figure 10. IMCs formed by interdiffusion of Al and Fe can be clearly observed in the TEM mapping images of Figure 10c–e. The chemical compositions at positions A and B in Figure 10c were 60.8 wt% Al/39.2 wt% Fe and 54.1 wt% Al/45.9 wt% Fe, indicating that the reaction layers near the Al alloy and steel were FeAl_3_ and Fe_2_Al_5_, respectively. The lack of Zn and oxide elements (≤0.1wt%) meant that the liquid Zn (melted by joule heating) was discharged to the edges of the nugget by the electrode force after acting as a flux for the liquid Al–Zn eutectic reaction that aided in removing the oxide layer from the Al alloy surface [44,45]. The average IMC thicknesses of the WO, CWB, and PWB samples were 4.2, 6.1, and 6.2 μm, respectively. The thickness of the IMC layer (δ) is a function of reaction time (*t*) and temperature (*T*), which is given by the following equation [46]:

$$\delta =\sqrt{2Kt},$$

where *K* is the growth factor ($K=K_{0}\exp\left(-Q/RT\right)$). According to the equation, an increase in the thickness of the reaction layer of adhesively bonded samples is probably due to a higher interfacial temperature arising from the high input current and limited current-carrying paths caused by the insulating characteristics of the adhesive. It should be noted that the joints with the adhesive showed locally formed pores and gas holes between the interfacial IMCs and Al alloy, as shown in Figure 10b. It is well known that the solubility of H in Al is extremely low at room temperature, and the H diffusion in liquid Al is as high as ~1 cm^2^/s [47]. Therefore, welding pores may form due to pyrolysis and vaporization of the epoxy-based adhesive residue during DSW resulting from volume shrinkage owing to the solidification of the molten Al alloy.

**Figure 11:**
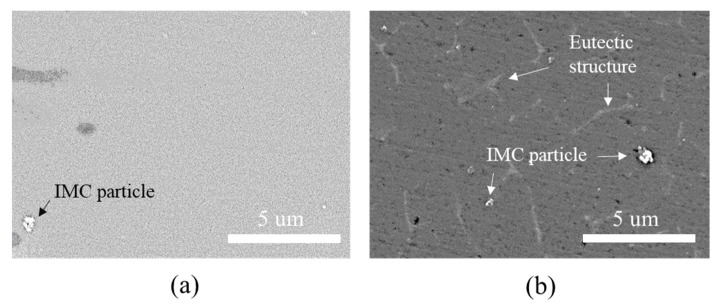
Microstructure of the welded zone of the Al6061 base metal (**a**) before and (**b**) after DSW.

The hardness profiles in Figure 9 show that the hardness in the welding area of the Al6061-T6 substrate decreased slightly when measuring approximately 4 mm from the matrix to the interface. The typical microstructures of the welded zone of the Al6061 base metal before and after DSW are shown in Figure 11. An irregular distribution of coarse intermetallic particles with a chemical composition of 51.9 wt% Al, 37.1 wt% Fe, 3.4 wt% Si, 3.1 wt% Cr, and 4.5 wt% Mg was observed throughout the as-received Al alloy. This particulate dispersion of secondary phases in the Al alloy leads to precipitation hardening [48]. On the other hand, in addition to the existing precipitated intermetallic particles, a eutectic Al–Si–Mg structure (89.1 wt% Al, 10.4 wt% Si, and 0.5 wt% Mg) was observed between the interdendritic spaces of the α-Al matrix after DSW (Figure 11b). It can be inferred that the large amount of input heat dissipates throughout the Al alloy base metal, inducing localized isothermal sections. A hardness reduction in the welded area and HAZ of Al6061-T6 alloy can be attributed to a microstructural transformation of the hard and thermodynamically unstable precipitates into a soft zone near the interface [49].

**Figure 12:**
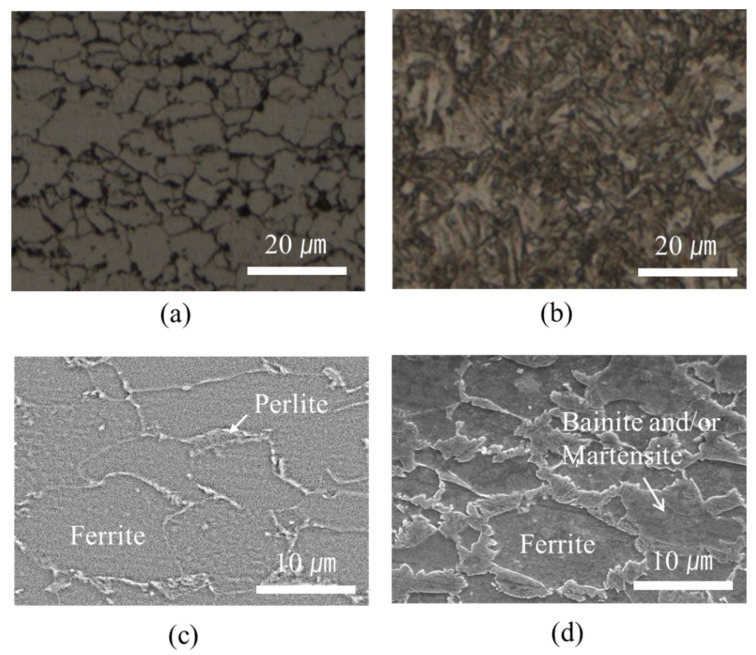
OM images of GA340YC steel (**a**) before and (**b**) after DSW; FE-SEM images of GA340YC steel (**c**) before and (**d**) after DSW.

In contrast, a markedly higher hardness of approximately 250 HV than that of the pristine base steel (180 HV) was observed in the welding area of the GA340YC steel (Figure 9). The microstructures of the GA340YC steel before and after DSW are shown in Figure 12. The as-received GA340YC consisted of approximately 10 μm α-Fe (ferrite) grains and pearlite in the grain boundaries, as shown in the FE-SEM image in Figure 12c. After DSW, the GA steel (Figure 12b) exhibited acicular IMCs. During DSW, the temperature can exceed 950 °C, at which α-ferrite transforms to γ-austenite. Subsequently, solidification by heat dissipation induces recrystallization and diffusionless phase transformations into acicular bainite or martensite phases, resulting in an increased hardness of GA steel. As clearly shown in Figure 12d, the welding area of GA340YC contained reduced α-ferrite grains and increased complex phases (bainite and martensite) throughout a large area. It should be noted that the adhesive-applied GA340YC steel had a wider area with a higher hardness than that of the GA340YC sample without adhesive.

**Figure 13:**
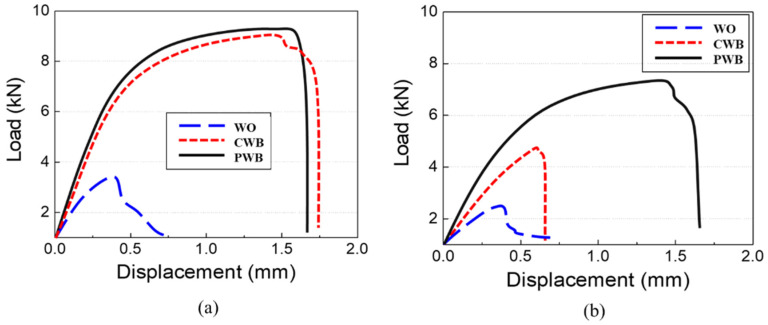
Force–displacement curves (**a**) before and (**b**) after 1500 h of SST.

Cross tensile tests of single lap joints were performed to evaluate the mechanical properties of the dissimilar joints. Figure 13a,b show the recorded force–displacement curves for the samples before and after 1500 h of SST, respectively. Despite the thicker interfacial IMC reaction layer and formation of gas holes at the interface, both adhesive-applied weldments showed much higher maximum strengths and strains compared to those of the WO sample due to the supplementary bond strength of the adhesive. The mechanical properties of the CWB sample were similar to those of the PWB sample before SST. However, after 1500 h of SST, the CWB sample showed significantly reduced maximum strength (48% reduction) and displacement values (63% reduction). In contrast, the PWB sample exhibited lower reduction (20% reduction in maximum strength and 5% reduction in displacement), as shown in Figure 13b, indicating that PLAT improved the long-term reliability under a corrosive environment. It can be inferred that removing the mechanically weak oxide and thin hydroxide layers from the surface of the Al6061-T6 alloy and replacing them with a new, uniform, and thin oxide layer resulted in a higher adhesive bond strength and improved the long-term reliability of the weldments in a corrosive environment.

**Figure 14:**
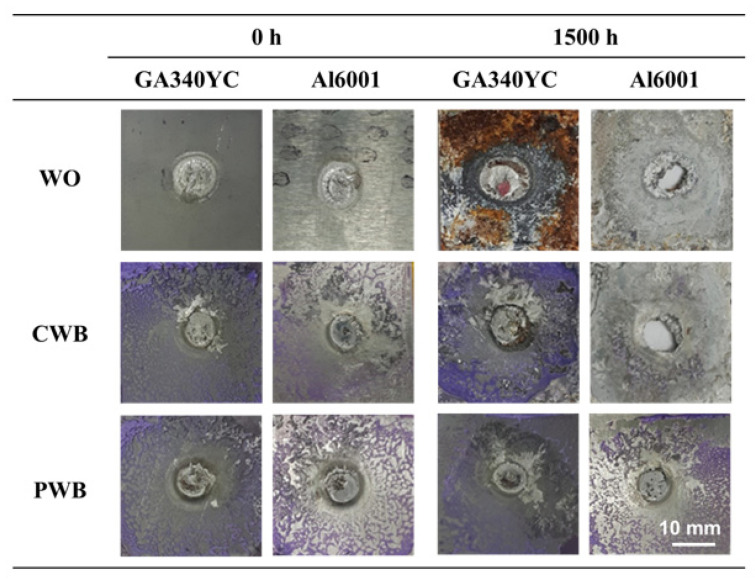
Appearance of fractured GA 440 and Al6061-T6 after shear tensile tests before and after 1500 h of SST.

**Figure 15:**
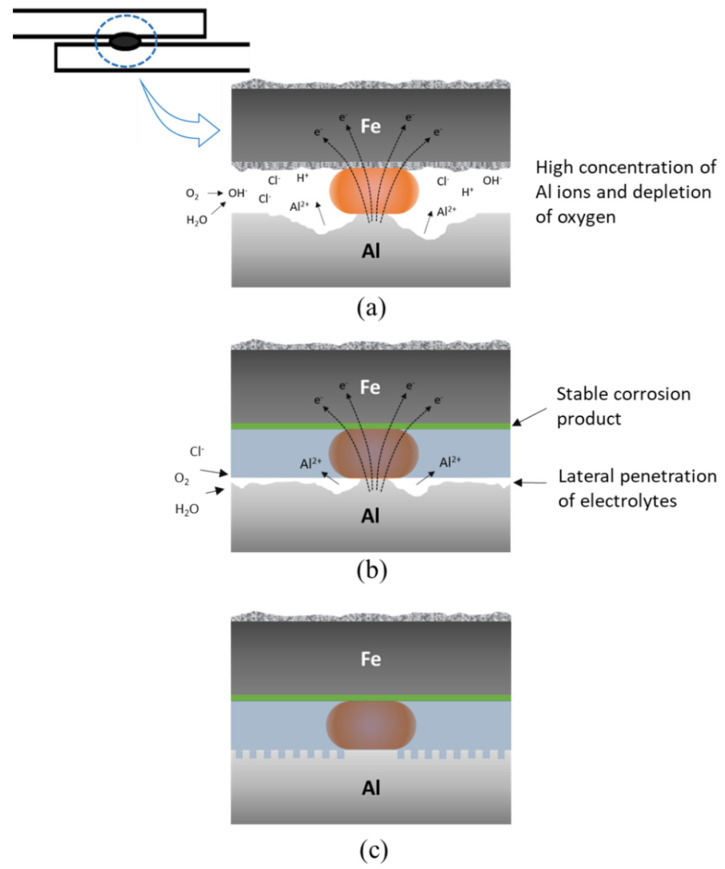
Schematics of observed failure in the (**a**) WO, (**b**) CWB, and (**c**) PWB samples.

To understand the joint failure behavior, the shapes of the fracture surfaces after shear tensile tests of each sample before and after 1500 h of SST were observed and are shown in Figure 14. Before the corrosion test, all joint specimens showed interfacial fracture due to the brittle interfacial Fe–Al reaction layer. In the joints with adhesive (CWB and PWB), a mixed adhesion/cohesive failure pattern was observed in which the adhesive remained on both sides of the adherends. It was clear that a larger area of adhesive remained on the Al alloy substrate of the PWB sample, which was attributed to the removal of the weakly bonded hydroxide layer and the increased contact area arising from the formation of a micro-rough surface by laser ablation. After 1500 h of SST, GA340YC in the WO sample had undergone severe galvanic and crevice corrosion, as schematically depicted in Figure 15a. A concentration of nonvolatile Al ions accumulated in the crevice on the micro-environmental level because of the continuous vaporization of Cl-containing water in the SST chamber. The pH thus decreased inside the crevice, and the highly acidic conditions due to deoxygenation resulted in an acceleration of corrosion with net anodic reactions occurring within the crevice [50].

Notably, a plug-type fracture was observed after shear tensile testing of the WO specimens. This may have occurred because the matrix surrounding the nugget experienced accelerated anodic dissolution due to localized electrochemical galvanic reactions, which would reduce the thickness of the base metal adjacent to the joint and subsequently weaken the mechanical properties of the Al6061-T6 alloy plate (schematically shown in Figure 15a). It should be emphasized that failure between the adhesive and Al6061 of the CWB sample was observed after 1500 h of SST, and, as shown in Figure 14, the adhesive was completely delaminated from the Al alloy substrate. This indicated that electrochemical erosion occurred via the lateral penetration of corrosive electrolytes from the edge along the adhesive/Al alloy interface. Thus, as schematically illustrated in Figure 15b, the occurrence of electrochemical corrosion reactions, dissolving Al at the anodic area between the adhesive and Al oxide, disrupts the chemical interactions and causes poor adhesion between the adhesive and Al alloy. Similar to that in the WO sample, plug-type fracture was also observed in the CWB joint. Contrarily, for the PWB specimen, it is clear from the images that the failure mode remained mainly as mixed adhesion/cohesive even after long-term exposure to salt spray fog for 1500 h. This was attributed to the improved interfacial interaction between the adhesive and PLATed Al6061-T6 surface induced by the removal of the surface Al hydroxide and/or hydrated Al oxide layers and the increased bonding area caused by laser irradiation, as schematically illustrated in Figure 15c. It is noteworthy that the adhesive/GA interface exhibits durable adhesion, in which the Zn–Fe coating acts as a sacrificial inhibitor by forming stable insulating corrosion products such as ZnO and simonkolleite to slow anodic dissolution [51]. The experimental results suggest that the surface chemistry and structure of the Al alloy play an important role in the long-term reliability of the dissimilar joints of Al6016-T6 and GA340YC steel plates. Furthermore, surface modification of the Al6061 alloy using PLAT can impede moisture intrusion and lateral diffusion of corrosive electrolytes.

## 4. Conclusions

In this study, the seal DSW method was employed to achieve improved weldability and mechanical properties of the dissimilar joint between Al6061-T6 aluminum alloy and galvannealed HSLA steel. The dissimilar metal weld joint in an overlap configuration suffered from severe galvanic and crevice corrosion in the presence of a corrosive electrolyte. The application of a structural adhesive induced the formation of a thicker interfacial IMC reaction layer and gas holes at the interface due to increased joule heating, but the mechanical properties were more favorable than the joint without the adhesive due to the supplementary adhesive bond strength. The long-term reliability of the mechanical properties of dissimilar welding in a corrosive environment was strongly dependent on the bond strength between the adhesive and Al6061 alloy substrate. Upon exposure to a corrosive environment, corrosive media infiltrates the adhesive/Al alloy substrate interface and disrupts the chemical interactions, resulting in poor adhesion and thus adhesive delamination. Moreover, the surrounding matrix of the nugget experiences accelerated anodic dissolution due to localized electrochemical galvanic reactions, which weakens the mechanical properties of the Al6061-T6 alloy plate. Pulsed excimer laser irradiation of the Al6061 alloy substrate removed the weakly bonded native Al oxide and hydroxide layers and thus altered the chemistry and topography of the substrate surface. This prevented electrolytes from penetrating the adhesive/Al alloy interface, which enhanced the long-term stability of the chemical interactions between the two materials. The results presented herein demonstrate excimer laser irradiation as a simple and environmentally friendly processing technology for establishing dissimilar metal bonding between Al6061 aluminum alloy and galvannealed HSLA steel with robust adhesion and long-term reliability.

## Figures and Tables

**Table 1:** Chemical composition of Al6061 Al alloy, 340YC steel and GA coating (wt%).

Materials	C	Mn	Si	P	S	Cu	Cr	Mg	Zn	Al	Fe
Al6061-T6	-	-	0.65	-	-	0.2	0.15	0.8	-	Bal.	0.2
340YC	0.09	1.0	0.25	0.025	0.01	-	-	-	-	-	Bal.
GA coating	-	-	-	-	-	-	-	-	Bal.	0.43	12.9

**Table 2:** Mechanical properties of Al6061 Al alloy and GA340YC steel plate.

Materials	TS (MPa)	YS (MPa)	El. (%)
Al6061-T6	335	270	12
GA340YC	425	364	25

**Table 3:** Welding conditions with which all weldments have similar nugget diameter.

Name	Process	Welding Condition (Pressure-Current-Time)	Notes
WO	Welding-only	2 kN-9 kA-250 ms	
CWB	Conventionally-cleaned Weld-Bonding	5 kN-14 kA-250 ms	Annealed at 180 °C for 30 min
PWB	PLATed Weld-Bonding	5 kN-14 kA-250 ms	

## Data Availability

Not applicable.
